# Topic Pages: *PLoS Computational Biology* Meets Wikipedia

**DOI:** 10.1371/journal.pcbi.1002446

**Published:** 2012-03-29

**Authors:** Shoshana J. Wodak, Daniel Mietchen, Andrew M. Collings, Robert B. Russell, Philip E. Bourne

**Affiliations:** 1 Hospital for Sick Children, Toronto, Canada; 2 Department of Biochemistry, University of Toronto, Toronto, Canada; 3 Department of Molecular Genetics, University of Toronto, Toronto, Canada; 4 EvoMRI Communications, Jena, Germany; 5 Open Knowledge Foundation Germany, Berlin, Germany; 6 Public Library of Science, Cambridge, United Kingdom; 7 Cell Networks, University of Heidelberg, Heidelberg, Germany; 8 Department of Pharmacology, University of California San Diego, La Jolla, California, United States of America; 9 Skaggs School of Pharmacy and Pharmaceutical Sciences, University of California San Diego, La Jolla, California, United States of America

While there has been much debate about the coverage and quality of Wikipedia (starting with an article in 2005 [1]), there is no doubt about its value (and increasing role) as a reference source and starting point for in-depth research. For example, within the biomedical sciences, there have been recent articles about the accuracy and completeness of drug information in Wikipedia [2], Wikipedia as a source of information in nursing care [3] and mental disorders [4], and making biological databases available through Wikipedia [5].

Is this the case for computational biology as well? Probably yes; however, at present our profession seems to gain more than it gives. We suggest a principal reason for this limited breadth and depth of coverage of topics in computational biology is one that affects a number of disciplines: reward. Authors in the biomedical sciences get academic reward for publishing papers in reputable journals that are indexed in PubMed and have associated digital object identifiers (DOIs). In contrast, contributions to Wikipedia can be anonymous and do not count for much in the current system of academic advancement. We hope to help to resolve this disparity in *PLoS Computational Biology*.

This month, we have published our first Topic Page on “Circular Permutations in Proteins” by Spencer Bliven and Andreas Prlić [6] as part of our Education section. Topic Pages are the version of record of a page to be posted to (the English version of) Wikipedia. In other words, *PLoS Computational Biology* publishes a version that is static, includes author attributions, and is indexed in PubMed. In addition, we intend to make the reviews and reviewer identities of Topic Pages available to our readership. Our hope is that the Wikipedia pages subsequently become living documents that will be updated and enhanced by the Wikipedia community, assuming they are in keeping with Wikipedia's guidelines and policies, either by individuals, or, perhaps as is already happening in medicine and molecular and cell biology, by something more organized, or with a more formal review structure. We also hope this will lead to improved scholarship in a changing medium of learning, in this case made possible by the Creative Commons Attribution License that we use.

Our Editorial Board has been enthusiastic in its support of this initiative and a number of Topic Pages are under development. We hope you will contribute too; please send ideas for Topic Pages to ploscompbiol@plos.org. We are looking for topics in computational biology that are of interest to our readership, the broader scientific community, and the public at large, *and* that are not yet covered, or only poorly so (i.e., exists as a “stub”), in Wikipedia: http://en.wikipedia.org/wiki/Wikipedia:WikiProject_Computational_Biology. Our guidelines for Topic Pages are available here: http://www.ploscompbiol.org/attachments/topicpages.pdf. Wikipedia is the world's most widely used knowledge source, and computational biology should be appropriately represented—please help. New uses of Wikipedia are being explored, as a recent example illustrates [7]. Who knows what you might be contributing to?
